# Towards a better understanding of arterial calcification disease progression in CKD: investigation of early pathological alterations

**DOI:** 10.1093/ndt/gfac301

**Published:** 2022-10-31

**Authors:** Geoffrey Van den Bergh, Britt Opdebeeck, Cédric Neutel, Pieter-Jan Guns, Guido De Meyer, Patrick D'Haese, Anja Verhulst

**Affiliations:** Laboratory of Pathophysiology, University of Antwerp, Wilrijk, Belgium; Laboratory of Pathophysiology, University of Antwerp, Wilrijk, Belgium; Laboratory of Physiopharmacology, University of Antwerp, Wilrijk, Belgium; Laboratory of Physiopharmacology, University of Antwerp, Wilrijk, Belgium; Laboratory of Physiopharmacology, University of Antwerp, Wilrijk, Belgium; Laboratory of Pathophysiology, University of Antwerp, Wilrijk, Belgium; Laboratory of Pathophysiology, University of Antwerp, Wilrijk, Belgium

**Keywords:** arterial media calcification, arterial stiffness, endothelium, nitric oxide

## Abstract

**Background:**

Cardiovascular disease remains the leading cause of death in chronic kidney disease (CKD) patients, especially in those undergoing dialysis and kidney transplant surgery. CKD patients are at high risk of developing arterial media calcifications (AMC) and arterial stiffness. We hypothesized that investigation of disease progression at an early stage could provide novel insights in understanding AMC etiology.

**Methods:**

An adenine diet was administered to male Wistar rats to induce AMC. Rats were sacrificed after 2, 4 and 8 weeks. AMC was measured by assessment of aortic calcium and visualized using histology. Arterial stiffness was measured *in vivo* by ultrasound and *ex vivo* by applying cyclic stretch of physiological magnitude on isolated arterial segments, allowing us to generate the corresponding pressure–diameter loops. Further, *ex vivo* arterial reactivity was assessed in organ baths at 2 and 4 weeks to investigate early alterations in biomechanics/cellular functionality.

**Results:**

CKD rats showed a time-dependent increase in aortic calcium which was confirmed on histology. Accordingly, *ex vivo* arterial stiffness progressively worsened. Pressure–diameter loops showed a gradual loss of arterial compliance in CKD rats. Additionally, viscoelastic properties of isolated arterial segments were altered in CKD rats. Furthermore, after 2 and 4 weeks of adenine treatment, a progressive loss in basal, nitric oxide (NO) levels was observed, which was linked to an increased vessel tonus and translates into an increasing viscous modulus.

**Conclusions:**

Our observations indicate that AMC-related vascular alterations develop early after CKD induction prior to media calcifications being present. Preventive action, related to restoration of NO bioavailability, might combat AMC development.

## Graphical Abstract

**Graphical Abstract:**
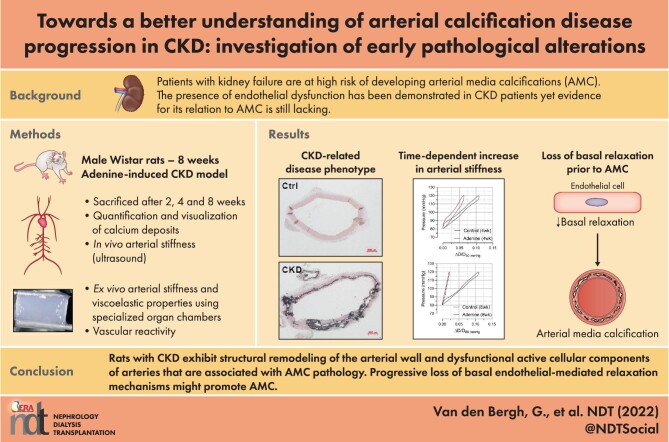

KEY LEARNING POINTS **What is already known about this subject?**

- Cardiovascular disease is still the leading cause of death in chronic kidney disease (CKD) patients, especially in those undergoing dialysis and/or kidney transplant surgery. Patients at every age in these groups are at high risk of developing arterial media calcifications (AMC) and arterial stiffness.
- The endothelium acts as the primary sensor of circulating pathological stimuli. Even though the presence of endothelial dysfunction has been demonstrated in CKD patients which are at high risk of developing AMC, evidence for a contributory role of endothelial cells to the pathophysiology of AMC is still lacking.

**What this study adds?**

- AMC in the adenine-induced CKD rat model was already initiated at an early timepoint Besides structural remodeling of the arterial wall (i.e. morphological disruption by the deposition of calcified lesions), dysfunctional active cellular components of arteries are associated with AMC pathophysiology.
- A progressively increasing vessel tonus due to a loss in basal nitric oxide resulted in an increased viscoelastic modulus.
- Progressive loss of basal endothelial–mediated relaxation mechanisms might promote AMC. This creates a situation in which excessive wall stiffness, due to increasing tonus or morphological changes, can no longer be compensated for by endothelial cells.

**What impact this may have on practice or policy?**

- It would be intriguing to know whether preserving/restoring endothelial function could be beneficial to combat AMC progression in CKD patients.
- The search for novel options to treat AMC, which has been mainly concentrated at the level of the vascular smooth muscle cell, may need to be expanded to intimal layer targets.
- Loss of arterial compliance early after the onset of renal function decline might play a crucial role in vascular outcome. Preventive measures to combat AMC might thus be warranted.

## INTRODUCTION

Cardiovascular disease remains the leading cause of death in chronic kidney disease (CKD) patients, especially in those undergoing dialysis and kidney transplant surgery [1, 2]. Almost a decade ago, the American Heart Association published a statement that individuals with CKD are at high risk for cardiovascular disease and must therefore be treated preventively [3]. Older adults with early stage CKD are 6-fold more likely to die from cardiovascular disease than to develop end-stage renal disease [4]. The pathophysiological mechanisms responsible for this increased cardiovascular risk in CKD patients are complex and multifactorial [5]. Traditional cardiovascular risk factors, such as hypertension, hyperglycemia and dyslipidemia, are highly prevalent in patients with CKD [6–8], although these parameters only account for a portion of the equation that describes the elevated cardiovascular risk in CKD patients [9].

Vascular aging is a non-traditional risk factor for CKD-related cardiovascular risk and is characterized by arterial media calcification (AMC). In a population of end-stage renal disease patients on hemodialysis the presence and extent of arterial calcification were strong predictors of cardiovascular and all-cause mortality [10]. Young dialysis patients are prone to increased cardiovascular mortality, equivalent to older adults in the general population without renal function loss [11], and very often develop AMC [12]. There is a general consensus that CKD causes a systemic, pro-inflammatory state that contributes to accelerated aging of the cardiovascular system and ultimately facilitates vascular and cardiac remodeling processes [13]. Indeed, a premature (vascular) ageing phenotype is observed in children on hemodialysis [12]. The progressive loss of renal function is accompanied by the retention of calcium, phosphate and uremic toxins that would normally be excreted in the urine. Both retained minerals and toxins are able to damage the endothelium [14] and vascular smooth muscle cells (VSMCs) [15]. VSMCs are considered to be the main type of cells driving arterial calcification, which is an actively regulated process [16]. The endothelium on the other hand acts as the primary sensor of circulating pathological stimuli. Even though the presence of endothelial dysfunction has been demonstrated in CKD patients [17–19], there is still a current lack of evidence for endothelial cells (ECs) to contribute to the pathophysiology of AMC. Recently our group demonstrated that ECs play a role in the development of AMC, of non-uremic origin, using the well-established warfarin model in mice and rats [20, 21]. Others have also shown that endothelial nitric oxide synthase (eNOS), the major enzyme responsible for endothelial nitric oxide production, is protective against arterial calcification in mice and rats [22–24].

Structural, pathological changes occurring during AMC such as the deposition of hydroxyapatite, the disruption of elastic lamellae and the deposition of collagen are associated with increased arterial stiffness by increasing the wall rigidity [25, 26]. AMC, by increasing stiffness of large central arteries, contributes to increased pulse wave velocity (PWV), early pulse wave reflection and elevated cardiac afterload [27]. Elevated cardiac afterload in its turn results in left ventricular hypertrophy (LVH), and early pulse wave reflection impairs coronary filling and cardiac function [28]. Arterial remodeling and premature aging of the arterial tree is already observed in early stages of CKD [29]. Lowering arterial stiffness is proven to be worthwhile for improving renal and cardiovascular prognosis [30]. Furthermore, while a decrease in renal function can increase arterial stiffness, increased arterial wall rigidity, in turn, causes a further decline in renal function thus creating a vicious cycle [31].

The adenine-induced CKD model is used extensively to investigate both the underlying mechanisms as well as possible treatments for CKD-induced AMC [32–36]. Chronic administration of adenine via the diet induces severe renal damage and as a result decline in renal functionality. When combined with dietary administration of extra phosphate, the model facilitates the development of AMC and LVH with preserved ejection fraction [37, 38]. This model strongly resembles human CKD patients with renocardiac syndrome [38, 39]. Our group has previously shown that renal function decline starts quickly after the onset of the adenine diet administration [36, 40, 41]. In the present manuscript, the grade of severe CKD-induced AMC with LVH was achieved by feeding rats an adenine rich diet for 4 weeks, followed by a high-phosphate diet for 4 weeks [35]. In order to facilitate a more thorough understanding of the early alterations in biomechanics and cellular functionality during AMC development, we investigated earlier (2 and 4 weeks adenine) timepoints as well.

## MATERIALS AND METHODS

### Animals

Housing conditions and experimental procedures were in accordance with the DIRECTIVE 2010/63/EU of the European Parliament and were approved by the local animal research authorities (Ethics Committee University of Antwerp, Permit: 2019-51). Forty-four male Wistar-Han rats (225–250 g; Charles-River Laboratories, Belgium) were held at the local animal facility with free access to water and chow. The rats were randomly assigned into different groups covering different timepoints (2, 4 and 8 weeks), consisting of (i) a control group with normal renal function and (ii) a CKD group that develops AMC. In the second group, CKD was induced by the administration of a high-phosphate (1.2%) diet (Ssniff, Spezialdiäten, Soest, Germany) for 2 weeks followed by a 0.75% adenine-rich diet (Ssniff, Spezialdiäten, Soest, Germany) for 4 weeks. Animals of the 2 and 4 weeks groups, respectively, were sacrificed after 2 and 4 weeks of adenine treatment. Subsequently, animals of the 8 weeks group were fed with the high-phosphate (1.2%) diet again for 4 weeks. The number of animals per timepoint is mentioned below each experiment in the figure legend (one animal of the 8 weeks group died prior to the start of the study). The schematic overview of the study setup is presented in Supplementary data A.

### Quantification and visualization of AMC

The total calcium content was measured in the aortic arch. At sacrifice the tissue was briefly rinsed with ice-cold 0.9% NaCl to get rid of excess blood and loose adherent tissue. All samples were stored at –20°C for further analysis. Calcium was measured by flame atomic absorption spectrometry as described elsewhere [20]. In short, prior to the measurement, tissues were wet weighed on a precision balance and digested in 65% nitric acid (HNO_3_) at 60°C overnight. Subsequently, the final volume of the aortic tissue digest was adjusted to 2.5 mL. Finally, samples were diluted with 0.1% La(NO_3_)_3_ to eliminate chemical interference during the measurement. Aortic calcium content was calculated and expressed as mg/g of wet tissue. Qualitative staining for calcified lesions was performed on formaldehyde fixed, paraffin-embedded transversal sections of the thoracic aorta by the Von Kossa method. Images were taken on a Leica DMR microscope (Leica Camera AG).

### Renal function

Renal function at each timepoint (2, 4 and 8 weeks) was evaluated by serum creatinine measurement according to the Jaffé method [42].

### Ultrasound measurement of arterial stiffness

Ultrasound imaging was performed in rats under anesthesia 2% (v/v) isoflurane (Forene; Abbvie, Belgium) using a high-frequency ultrasound system (Vevo2100, VisualSonics). Body temperature was maintained at 36–37°C and heart rate was kept at 400 ± 50 beats/min. Abdominal aorta PWV (aPWV) was determined according to the method developed by Di Lascio *et al*. with a 24-MHz transducer [43]. Briefly, pulse-wave Doppler was used to measure abdominal aortic flow velocity (V). Aortic diameter (D) was measured using B-mode images of the abdominal aorta in ECG-gated Kilohertz Visualization (EKV) imaging mode. The ln(D)-V loop method was then applied to calculate aPWV, using MathLab v2014 software (MathWorks). Abdominal aortic distensibility was calculated based on EKV images using the VevoVasc software (VisualSonics).

### Biomechanical and aortic reactivity evaluation of isolated arterial segments

Rats were euthanized by perforating the diaphragm, under anaesthesia (pentobarbital sodium, 150 mg/kg i.p.). The left carotid artery was carefully excised, stripped of adherent tissue and subdivided into segments of ±1.5 mm using a calibrated stereomicroscope. Immediately after excision, carotid segments were immersed in Krebs Ringer (KR) solution, which serves as a buffer solution and was maintained at pH 7. This physiological buffer contained (in mM): NaCl 118, KCl 4.7, CaCl_2_ 2.5, KH_2_PO_4_ 1.2, MgSO_4_ 1.2, NaHCO_3_ 25, CaEDTA 0.025 and glucose 11.1. During the whole experiment KR solution was aerated with 95% O_2_/5% CO_2_ and kept at 37°C.

To study the biomechanical properties (i.e. arterial stiffness) of carotid artery segments at physiological pressure and frequency, the Rodent Oscillatory Tension Set-up to study Arterial Compliance (ROTSAC) was used as previously explained [20, 44]. The Peterson's elastic modulus (Ep) was calculated as an *ex vivo* measure of arterial stiffness. The Ep was measured at baseline in KR solution and following maximal contraction. The latter was induced by adding a combination of phenylephrine (2 μM, PE, an α1-adrenergic receptor agonist) and N(ω)-nitro-L-arginine methyl ester (300 μM, LN, a pan-NOS inhibitor,) to the organ bath. Statistical analysis was performed for Ep at a physiological pressure range (80–120 mmHg). Additionally, Ep was evaluated at different (mean) pressures (with increments of 20 mmHg) while keeping the pulse pressure constant.

Furthermore, pressure–diameter (PD) loops of were constructed. The total PD relationship of an arterial segment can be separated in a viscous, an elastic and an inertial component. Quantification of both the elastic and viscous modulus was performed as described elsewhere [45–48]. Briefly, the following PD equation was used to calculate the viscous and elastic modulus:

$$\begin{equation*}{P}_{total}\ \left( t \right) = \ {E}_{pd}D\left( t \right) + \mu \frac{{dD\left( t \right)}}{{dt}} + M\frac{{{d}^2D\left( t \right)}}{{d{t}^2}}\end{equation*}$$

Where P_*total*_ is the overall PD relationship, E_*pd*_ the elastic modulus, μ the viscous modulus and M the inertial modulus. For each pulse, the area of the loop hysteresis was reduced by iterating both μ and M, manually, until the minimum loop area was obtained. The value of μ and M at the minimum hysteresis area were considered equivalent to the viscous and inertial modulus, respectively. Then, an exponential function was fitted on the resulting pure elastic PD relationship. The slope of that function, at the mean pressure of 100 mmHg, is the E_*pd*_.

Arterial reactivity was studied at the 2- and 4-week timepoints by isometric contraction and relaxation upon pharmacological stimulation on two left carotid artery segments of each animal, LN (300 μM) was added to the organ bath for one segment at the beginning of the experiment to inhibit all NOS activity. The isometric Statham UC2 force transducer (Gould, USA) was used to measure force generation by the VSMCs while holding the segment at a constant muscle length. The contractile force is reported in milliNewton (mN). After placing the carotid rings, the segments were stretched until a preload of 17 mN was attained. Next, a concentration response curve of PE (range of 3 nM to 10 μM) was executed. This was followed by endothelium-dependent relaxation of the contracted tissue using acetylcholine (ACh) (range of 3 nM to 10 μM) that results in the activation of eNOS synthase.

### Statistical analysis

Data were analyzed using Prism 8.4 (GraphPad). For multiple comparisons, ordinary one-way ANOVA with Tukey multiple comparison test was performed. For comparison between two treatment groups, a two-tailed Mann–Whitney U test was used to calculate the statistically significant differences. For testing the treatment factor in combination with other categorical factors (concentration–response curves), two-way ANOVA with Bonferroni correction was used. A *P*-value of ≤.05 was considered significant (95% confidence level).

## RESULTS

### Adenine-treated rats develop a CKD-related cardiovascular disease phenotype

**Figure 1:**
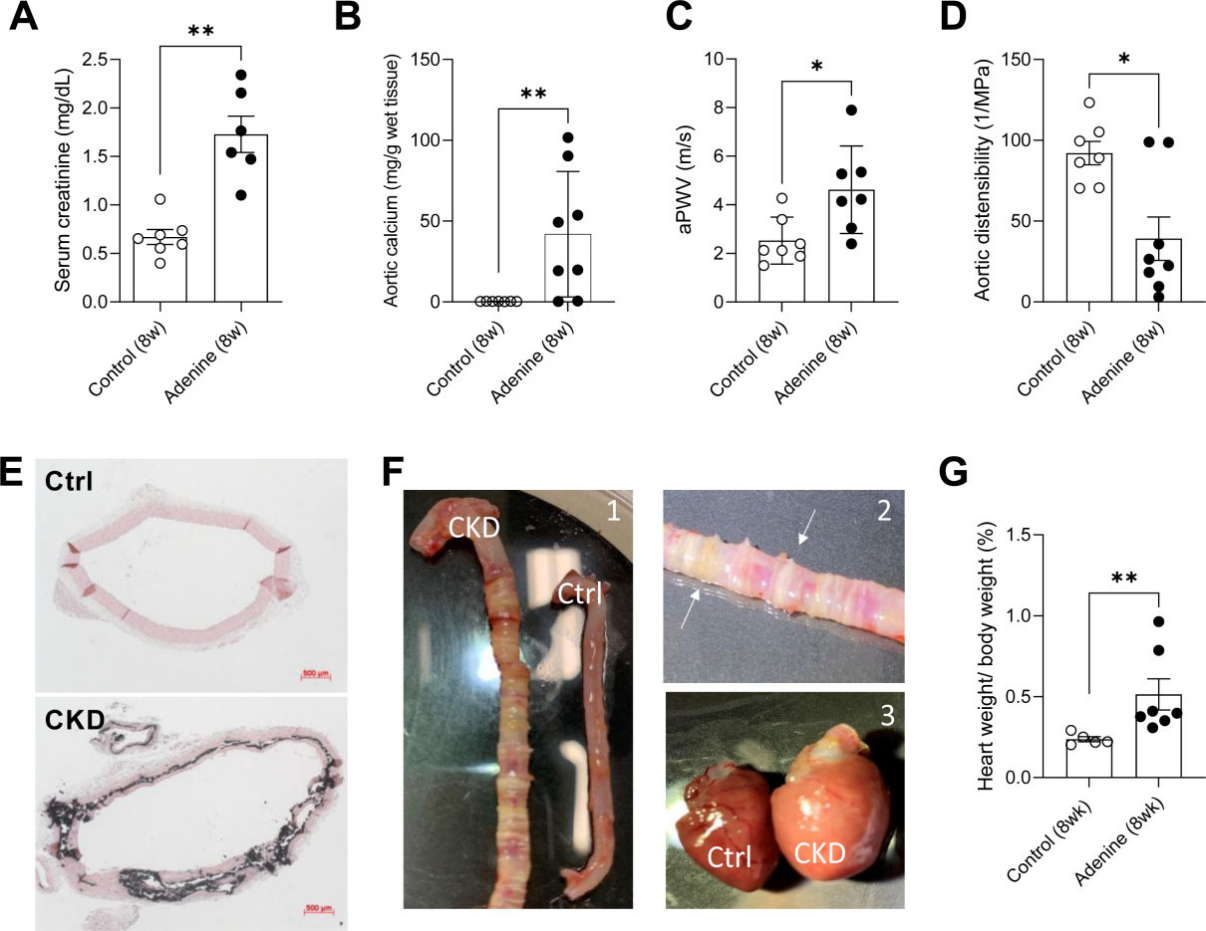
Adenine-treated rats develop a CKD-related cardiovascular disease phenotype. Serum creatine (**A**), aortic calcium (**B**), aPWV (**C**) and aortic distensibility (**D**) are shown for control and adenine-treated rats after 8 weeks. Excessive media calcification was confirmed by the Von Kossa method in CKD rats but not for controls (Ctrl) (**E**). (**F**) Macroscopic pictures of CKD and Ctrl tissue were taken side by side at the time of sacrifice to appreciate the dimensional changes that occurred during the 8-week treatment period: aorta (F1), magnified view of aorta, arrows point at ring-like mineralization (F2), heart (F3). Both the aorta and heart of adenine-treated (CKD) rats were visually enlarged after 8 weeks of treatment. At sacrifice the heart was removed and weighed on a precision balance. Next, obtained values were normalized against body weight (**G**). Mann–Whitney U test (two-tailed) (A–D, G): *P < .05; **P < .01. Data represented as mean ± SEM.

After 8 weeks of treatment with an adenine- and/or phosphate-enriched diet, serum creatinine was significantly increased in the treatment group, compared with controls (Fig. 1A). Furthermore, aortic calcium was significantly increased (Fig. 1B). AMC was confirmed, as large Von Kossa positive lesions were visible (Fig. 1E) in the macroscopically enlarged aorta (Fig. 1F). AMC was accompanied by elevated arterial stiffness, as evidenced by aPWV and distensibility by *in vivo* ultrasound (Fig. 1C and D). Rats who were given the adenine–phosphate enriched diet also had enlarged hearts (after 8 weeks) and a significantly elevated heart/body weight ratio, an indication of LVH (Fig. 1G). Control rats did not show any dimensional changes to the heart (Fig. 1F).

### Adenine-treated rats develop renal dysfunction prior to media calcifications and increased stiffness being present

**Figure 2:**
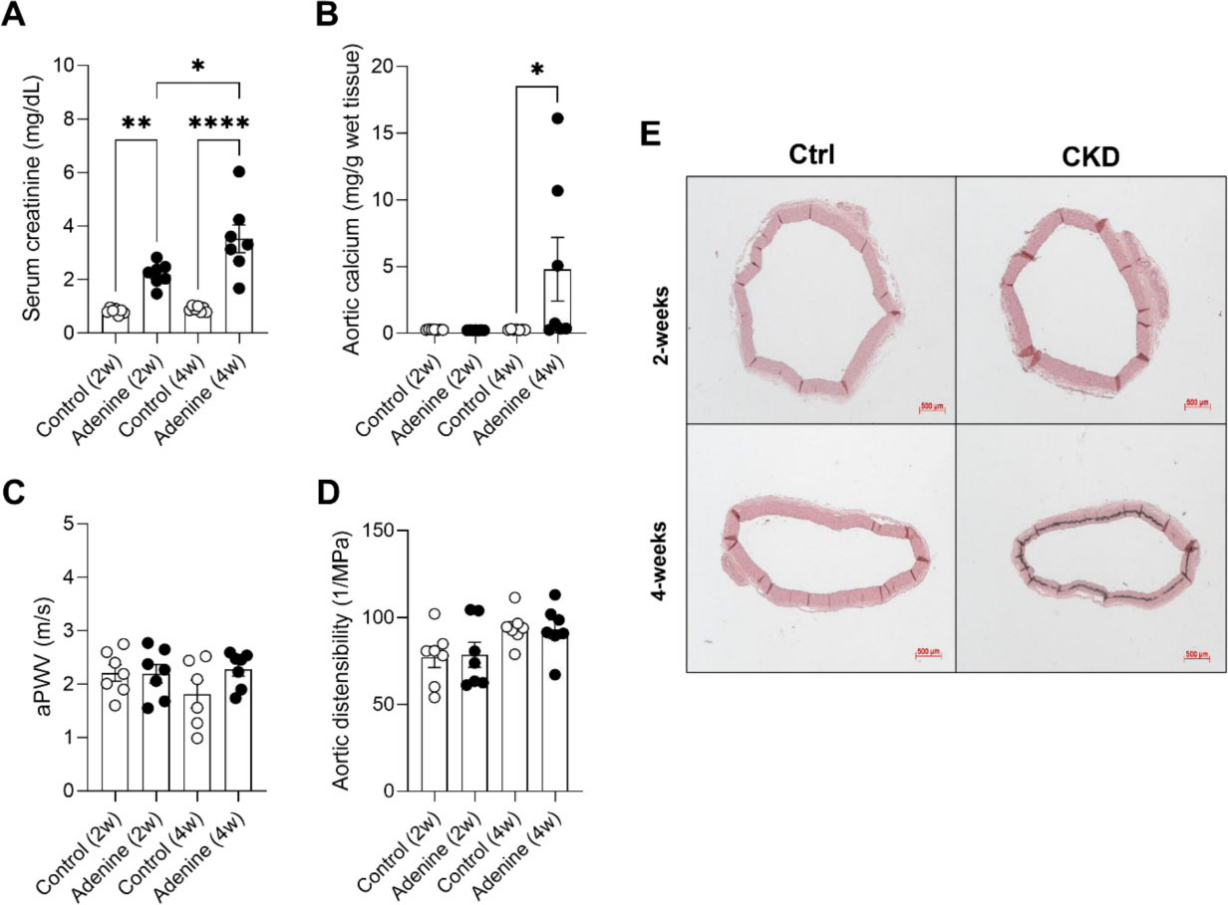
Adenine-treated rats develop renal dysfunction prior to media calcifications and increased *in vivo* stiffness being present. Serum creatinine (**A**) and total aortic calcium (**B**) are shown for each timepoint (2 and 4 weeks). Abdominal aPWV (**C**) and distensibility (**D**), measured by ultrasound, are shown for each timepoint (2 and 4 weeks). Number of animals per group (A–D): control 2 and 4 weeks: *n* = 7; adenine 2 and 4 weeks: *n* = 7. Ordinary one-way ANOVA with Bonferroni multiple comparison test (A–D): *P* > .05: not significant, not shown; *P < .05; **P < .01;****P < .0001. Data in bar graphs are represented as mean ± SEM. Formalin fixed, paraffin-embedded thoracic aorta cross sections (50× magnification) are used to visualize mineralization by the Von Kossa method (**E**). Calcium phosphate depositions (Von Kossa positive) are depicted as black precipitation. Cross sections are separated per condition (Ctrl, CKD) and per timepoint (2 and 4 weeks). Number of animals per group (E): control 2 and 4 weeks: *n* = 7; adenine 2 and 4 weeks: *n* = 7. Scale bar: 500 μm.

After 2 weeks of adenine treatment, rats possessed a significantly elevated serum creatinine concentration. Serum creatinine increased even more after 4 weeks and persisted over the course of the study versus time-matched controls. (Fig. 2A). There was no significant difference in total calcium content in the aortic arch after 2 weeks of adenine treatment compared with their controls, while after 4 weeks a significant increase was observed (Fig. 2B). Also, when visualizing AMC with Von Kossa staining on aortic tissue sections, the first signs of media calcifications appeared only after 4 weeks of adenine administration (Fig. 2E). AMC in the 4 weeks group, unlike in the 8 weeks group, was not yet accompanied by a significant increase in aPWV or aortic distensibility (Fig. 2C and D).

### Adenine-enriched diet induces a time-dependent increase in *ex vivo* arterial stiffness

**Figure 3:**
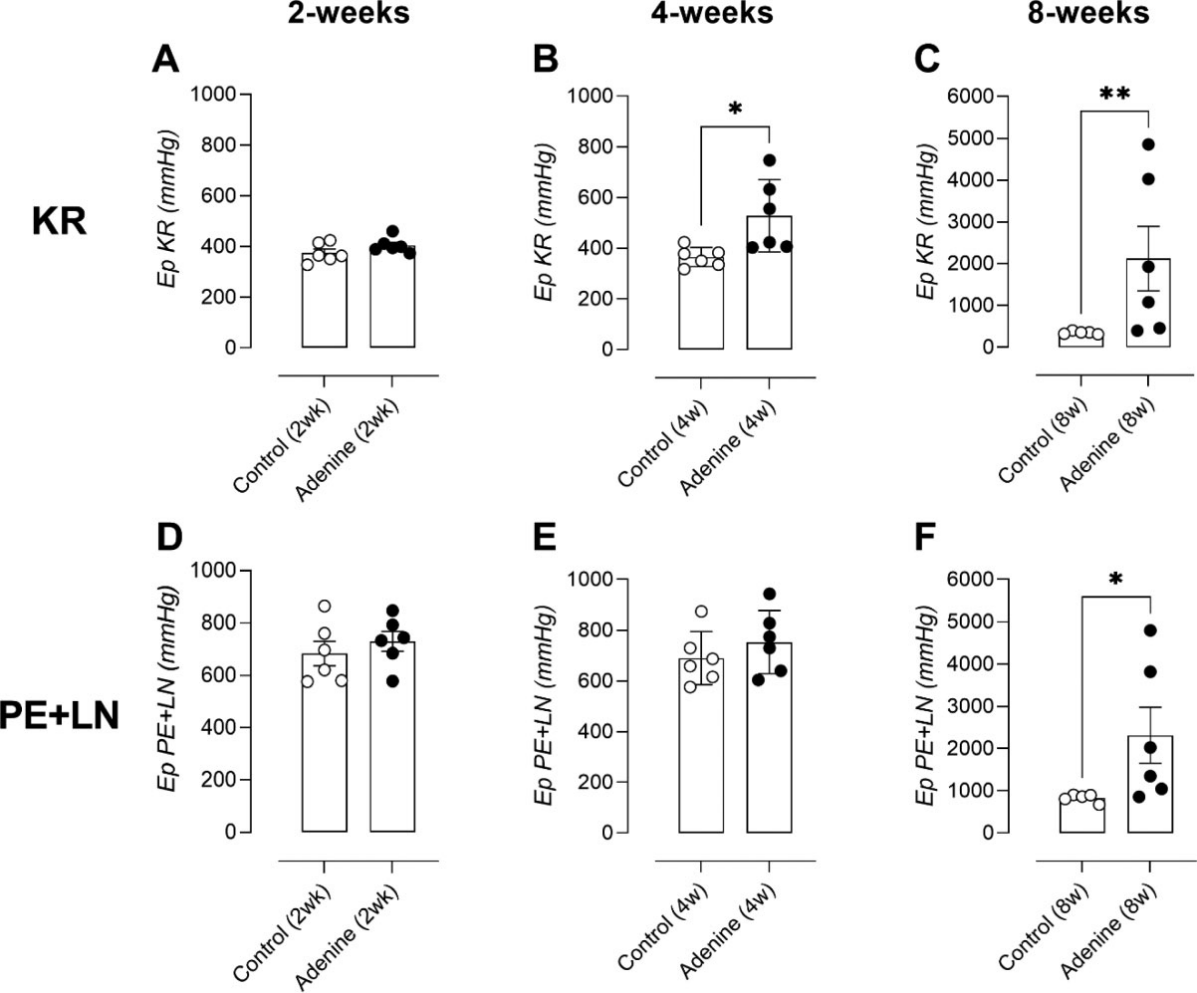
Adenine-enriched diet induces a time-dependent increase in *ex vivo* arterial stiffness. Biomechanical assessment of arterial stiffness. *Ex vivo* arterial stiffness, calculated by the Peterson's elastic modulus (Ep), is both shown in regular Krebs (KR) (**A**–**C**) and at maximal contraction (PE + LN) (**D**–**F**) split per timepoint (2, 4 and 8 weeks). Number of animals per group (A–F): control 2 and 4 weeks: *n* = 6; control 8 weeks: *n* = 5; adenine 2, 4 and 8 weeks: *n* = 6. Mann–Whitney U test (two-tailed) (A–F): *P* > .05: ns; *P < .05; **P < .01. Data in bar graphs represented as mean ± SEM.

After 2 weeks of adenine administration, arterial stiffness did not significantly differ from that of control arteries (Fig. 3A and D). In regular KR, adenine-treated rats possessed a significantly elevated Ep after 4 weeks (Fig. 3B), which disappeared after maximally contracting the segments with 2 μM phenylephrine (Fig. 3E). A large increase in Ep was observed after 8 weeks of adenine treatment, while control rats maintained their biomechanical properties (Fig. 3C). Moreover, after maximally contracting the artery segments a significant increased Ep was still observed (Fig. 3F).

### Adenine treatment alters the *ex vivo* viscoelastic properties of carotid arteries

**Figure 4:**
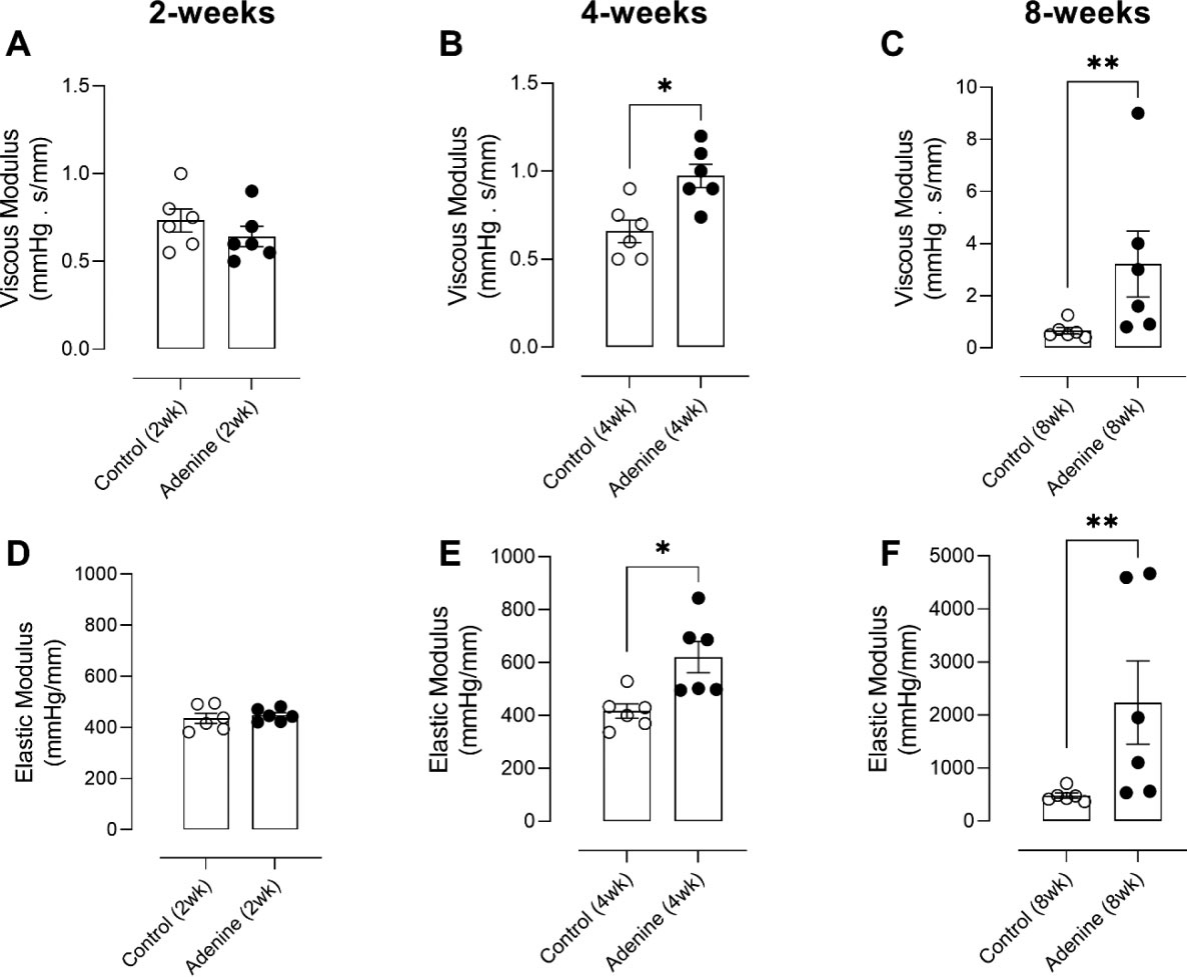
Adenine treatment alters the *ex vivo* viscoelastic properties of carotid arteries. Both the viscous and elastic moduli are unaltered after 2 weeks of adenine administration (**A, D**). The viscous modulus was significantly increased after 4 (**B**) and 8 (**C**) weeks of treatment. Additionally, the elastic modulus was also increased after 4 (**E**) and 8 weeks (**F**) in the adenine-treated animals. Mann–Whitney U test (two-tailed) (A–F): *P* > .05: not significant, not shown; *P < .05; **P < .01. Data in bar graphs represented as mean ± SEM.

**Figure 5:**
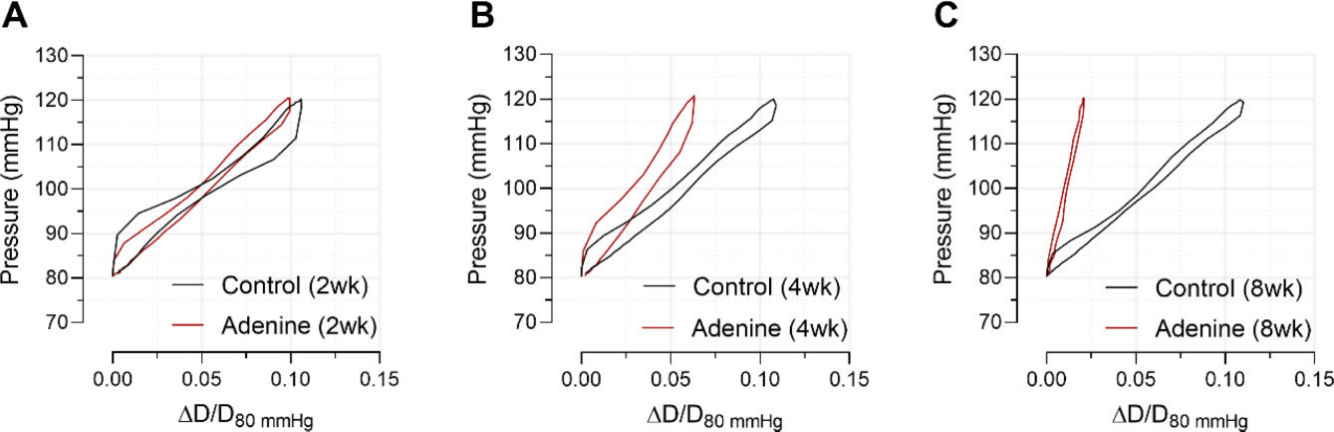
Representative tracings of *ex vivo*–acquired PD loops of rat left carotid artery segments after 2, 4 and 8 weeks. After 2 weeks (**A**), no clear difference was visible between PD loops obtained from left carotid arteries from adenine treated rats, compared with arteries from control rats. After 4 weeks, a noticeable ‘left shift’ of the PD loop was visible of arteries from adenine-treated rats, indicating an increased rigidity of the artery segment (**B**). After 8 weeks, a clear left shift of the PD loop of artery segments is observed in the adenine-treated rats, indicating a further increase in tissue stiffness (**C**). ΔD = D – D_80_ mmHg.

After 2 weeks of adenine diet, both the viscous and elastic modulus of the artery segments were unaltered (Fig. 4A and D). However, after 4 weeks, the viscous modulus was significantly increased (0.66 ± 0.06 vs 0.97 ± 0.07 mmHg.s/mm) (Fig. 4B). Also, the elastic modulus was significantly increased after 4 weeks of adenine administration (417 ± 27 vs 620 ± 59 mmHg/mm) (Fig. 4E). After 8 weeks of treatment, significant increases in both the viscous (0.66 ± 0.13 vs 3.22 ± 1.26 mmHg.s/mm) and elastic modulus (481 ± 49 vs 2236 ± 787 mmHg/mm) were observed (Fig. 4C and F). Representative tracings of *ex vivo*–acquired PD loops of rat left carotid artery segments after 2, 4 and 8 weeks of adenine supplementation are drawn to appreciate the increasing viscous and elastic moduli over the course of the study (Fig. 5).

**Figure 6:**
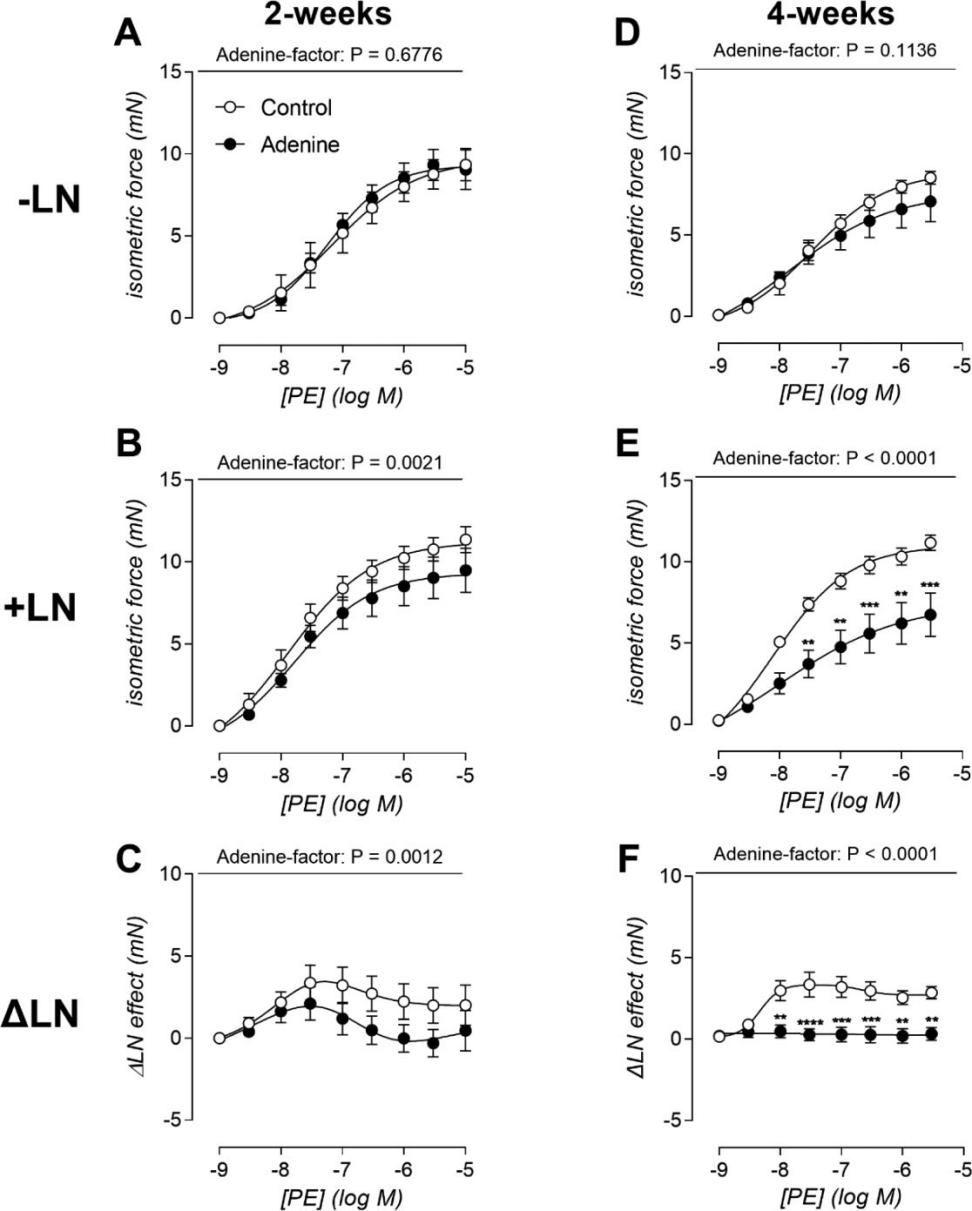
Loss of endothelial basal NO activity, prior to the presence of media calcifications. Aortic contractility, assessed by the addition of phenylephrine (PE, 3 nM–10 μM) to the organ bath. Two weeks: the concentration–response curve for PE is shown in the absence of N(ω)-nitro-L-arginine methyl ester (–LN) (**A**) and in the presence of LN (+LN) (**B**). Additionally, the LN effect was calculated (**C**). Four weeks: identical to previous timepoint (**D**–**F**). Number of animals per group (A–F): –LN, 2 weeks control: *n* = 6; –LN, 4 weeks control: *n* = 5; +LN, 2 and 4 weeks control/adenine: *n* = 6; ΔLN, 2 weeks control/adenine: *n* = 6; ΔLN, 4 weeks control/adenine: *n* = 5. Two-way ANOVA with Bonferroni multiple comparison test (A–F): *P* > .05: not significant, not shown; **P < .01; ***P < .001; ****P < .0001. Data represented as mean ± SEM.

### Loss of endothelial basal NO activity, prior to the development of media calcifications

Next to arterial stiffness, arterial reactivity was also evaluated by means of an *ex vivo* organ bath setup using left carotid artery segments. In the absence of LN, concentration–response curves for PE were similar for both groups after 2 and 4 weeks of adenine treatment (Fig. 6A and D). In contrast, when LN was added to the organ bath, the concentration–response curves of the control segments shifted up and left, while this was much less the case for the segments of the adenine-fed rats (adenine effect: *P* = .0021 in the 2-week group) (Fig. 6B). The response to LN (ΔLN) in the adenine rats that were treated for 2 weeks was significantly smaller (adenine effect: *P* = .0012) in segments of adenine fed rats compared with timepoint-matched controls (Fig. 6C). Attenuated contraction persisted and became more pronounced after 4 weeks of adenine treatment (Fig. 6E). This finding was also visualized by plotting the ΔLN effect, which after 4 weeks of adenine treatment shows a flatline for segments of adenine-fed rats. The sensitivity towards PE-induced VSMC contraction was unaltered after 2 and 4 weeks of treatment, as indicated by a similar EC50 value. This was also the case in the presence of LN (Supplementary data, B and C). These results indicate that any capability of these segments to relax against a contracting stimulus of PE (basal, unstimulated NO) was completely abolished. In contrast, ACh-mediated relaxation was not significantly altered after 2 weeks (*P* = .6312) and 4 weeks (*P* = .1938) of adenine treatment (graphs not shown).

## DISCUSSION

In the present study, administration of an adenine-enriched diet resulted in increased serum creatinine levels, development of AMC and a visually enlarged heart, mimicking human CKD patients with renocardiac syndrome [38, 39]. A significant rise in serum creatinine preceded the presence of any sign of elevated aortic calcium in our model. Since adenine treatment was stopped after 4 weeks and switched to the high-phosphate diet, creatinine values in the 8 weeks group were significantly lower compared with the 4 weeks group (values comparable to 2-week adenine treatment). Nevertheless, the largest calcified lesions and most pronounced arterial stiffness appeared at the end of the study (when the adenine diet was already stopped), suggesting that early loss of renal function might play a crucial role in vascular outcome. We observed large calcified lesions, disrupting aortic morphology, in visibly enlarged aortic cross-sections after 8 weeks of treatment. These changes were accompanied by significantly increased local arterial stiffness, which was measured at the abdominal aorta using ultrasound and on isolated carotid artery segments by quantifying Ep. Inclusion of earlier timepoints (2 and 4 weeks) in the study allowed us to present AMC onset at 4 weeks: the first signs of mild calcified lesions appeared after 4 weeks, while still absent at 2 weeks. The 2-week timepoint, however, allowed us to investigate early changes in cellular reactivity of arterial segments, possibly contributing to AMC.

To the best of our knowledge, this is the first time that *ex vivo* viscoelastic properties have been linked to cellular reactivity of arterial segments in a uremic CKD model with progressively increasing AMC and arterial stiffness. Previous findings regarding the presence of arterial stiffness in this model were confirmed and built upon: Nguy and colleagues [49], albeit having used different protocol of less concentrated adenine dosing, showed that adenine-induced renal failure negatively affected aortic compliance by reducing the rate of contraction and relaxation in aortic segments, translating into elevated arterial stiffness *in vivo* [50]. While in our model, *in vivo* PWV, a measure of arterial stiffness, was not significantly altered after 4 weeks of adenine diet, an increase in *ex vivo* carotid artery stiffness was already observed. Our *ex vivo* stiffness results suggest that the arterial segments of the adenine-treated groups possessed increased VSMC tonus.

When further dissecting the Ep in both a viscous and elastic component, a clear effect was seen on both. The viscous modulus, which is largely affected by VSMC tonus [46, 51, 52], was significantly increased in the adenine groups after 4 and 8 weeks. The endothelium has been linked to arterial wall viscosity by regulating arterial tonus in humans through NO production [53]. Leloup and colleagues previously showed that a lack of basal, unstimulated eNOS activity increases VSMC tonus [54]. When eNOS activity is lowered, VSMC tonus and arterial wall viscosity increase, leading to an increased tissue stiffness. However, when eNOS function is blocked *ex vivo*, by the addition of LN in the organ baths, differences in eNOS activity between groups are eliminated. Therefore, if the *ex vivo* stiffness was higher in the treatment group (at 4 weeks) before LN, but equal after LN, a possible involvement of altered (i.e. lowered) eNOS activity can be speculated. Interestingly, Yamada and colleagues [55] previously showed that the antioxidant Tempol was able to attenuate AMC in uremic rats, using the same adenine concentration as the one applied in the present study. Tempol is a well-known mimetic of superoxide dismutase with antioxidant properties that improves NO bioavailability and vascular function [56–58]. Since we have used the same animal model of AMC, an unreported effect of Tempol in the Yamada study might have been the restoration of vascular NO bioavailability, alongside its antioxidant effect. Future research investigating the direct effect on AMC of restoring NO bioavailability is worth carrying out.

Control rats maintained their biomechanical properties across all timepoints: the overall tissue stiffness, viscous and elastic moduli were not different between arterial segments of controls at different timepoints. At 8 weeks, such a large difference in Ep between groups could not be normalized by maximally contracting the segments. Therefore, arterial reactivity was not investigated for the last timepoint. Adenine-treated carotid segments showed an attenuated contraction (albeit not significant) versus controls after 4 weeks of adenine treatment. This difference between control segments and segments from adenine rats was further amplified after the addition of LN to the organ bath: it became apparent that adenine treatment was able to fully negate the effects LN usually has on contracted carotid segments as seen in the control group. Loss of basal, unstimulated NOS activity (i.e. failing to counteract against an imposed contraction by the addition of PE) and increased VSMC tonus go hand in hand, as seen in the absence of any LN effect and significantly higher Ep after 4 weeks of adenine treatment. A time-dependent, concurrent loss of contractile markers can be speculated in the CKD groups. Osteogenic transdifferentiation could be a possible explanation, next to loss of basal NOS activity, for the severely attenuated PE contraction we observed after 4 weeks.

Segments of rats treated for 2 weeks already showed an attenuated response to LN after 2 weeks, prior to calcifications being visually present, even though arterial wall viscosity was not altered at this timepoint. As expected, after 2 and 4 weeks, basal eNOS activity in control segments was completely inhibited after pan-NOS inhibition, which shifted the concentration curve up and left. Remarkably, though it seems to be counterintuitive at first sight, stimulated NO release by ACh administration was not significantly attenuated after 2 and 4 weeks of treatment, suggesting that only basal NO production was affected, while the receptor stimulated NO response was still intact in our model. Nguy *et al*. previously published that adenine-treated animals possessed no significant alterations in the sensitivity or maximal vasodilatory response to ACh even though these rats displayed all the characteristics of uremia [49]. Also, compromised basal, unstimulated NO release together with an intact receptor-stimulated NO response has been observed before in aortic segments of Fbn1C1039G/+ (Marfan model) mice [59], apolipoprotein E–deficient mice [60], stroke-prone spontaneously hypertensive rats [61] and warfarin-fed DBA/2J mice [20]. Results of the present paper further exemplified these differences between basal and stimulated vasodilator NO activity in vascular diseases.

Earlier we showed that endothelial cells are likely involved in the pathophysiology of warfarin-mediated (non-uremic) AMC in mice [20]. In the current manuscript, by using a biomechanical approach on isolated arterial segments, we confirm that endothelial (dys)function might be linked to AMC in uremic rats. Taken together, renal dysfunction was present at the 2-week timepoint, which was prior to obvious AMC development. Our observations indicate that the development of vascular pathology in the adenine-induced CKD model was already initiated at that early timepoint. It became clear that, besides structural remodeling of the arterial wall (i.e. morphological disruption by the deposition of calcified lesions), dysfunctional active cellular components of arteries might play a role in the pathophysiological mechanisms by which AMC is induced in CKD rats. Namely, a progressive loss in endothelial basal NO production, linked to an elevated vessel tonus, which translates into an increasing viscous modulus seems to be associated with the development of AMC. Conceivably, progressive loss of endothelial-mediated relaxation mechanisms might force VSMC into a pro-calcifying state in which excessive wall stiffness, due to increasing tonus or morphological changes, can no longer be compensated for by endothelial cells [62]. Failure to maintain a physiological VSMC tonus could promote changes in signaling pathways involved in sensing cellular and matrix stiffness which, ultimately, will change the fate of the VSMC phenotype [63, 64]. As such, the search for novel options to treat AMC, which has been mainly concentrated at the level of the VSMC, may need to be expanded to intimal layer targets. Moreover, preventive action, related to restoration of NO bioavailability, might combat AMC development.

## Supplementary Material

gfac301_Supplemental_File

## Data Availability

The data underlying this research will be shared upon reasonable request to the corresponding author.
