# Supplementary material for: Towards a better understanding of arterial calcification disease progression in CKD: investigation of early pathological alterations
Source: Nephrol Dial Transplant. 2022 Oct 31;38(5):1127–38. doi: 10.1093/ndt/gfac301 (PMC10320369; doi:10.1093/ndt/gfac301)
Supplement: gfac301_Supplemental_File [file gfac301_supplemental_file.docx]

**Supplemental Data**


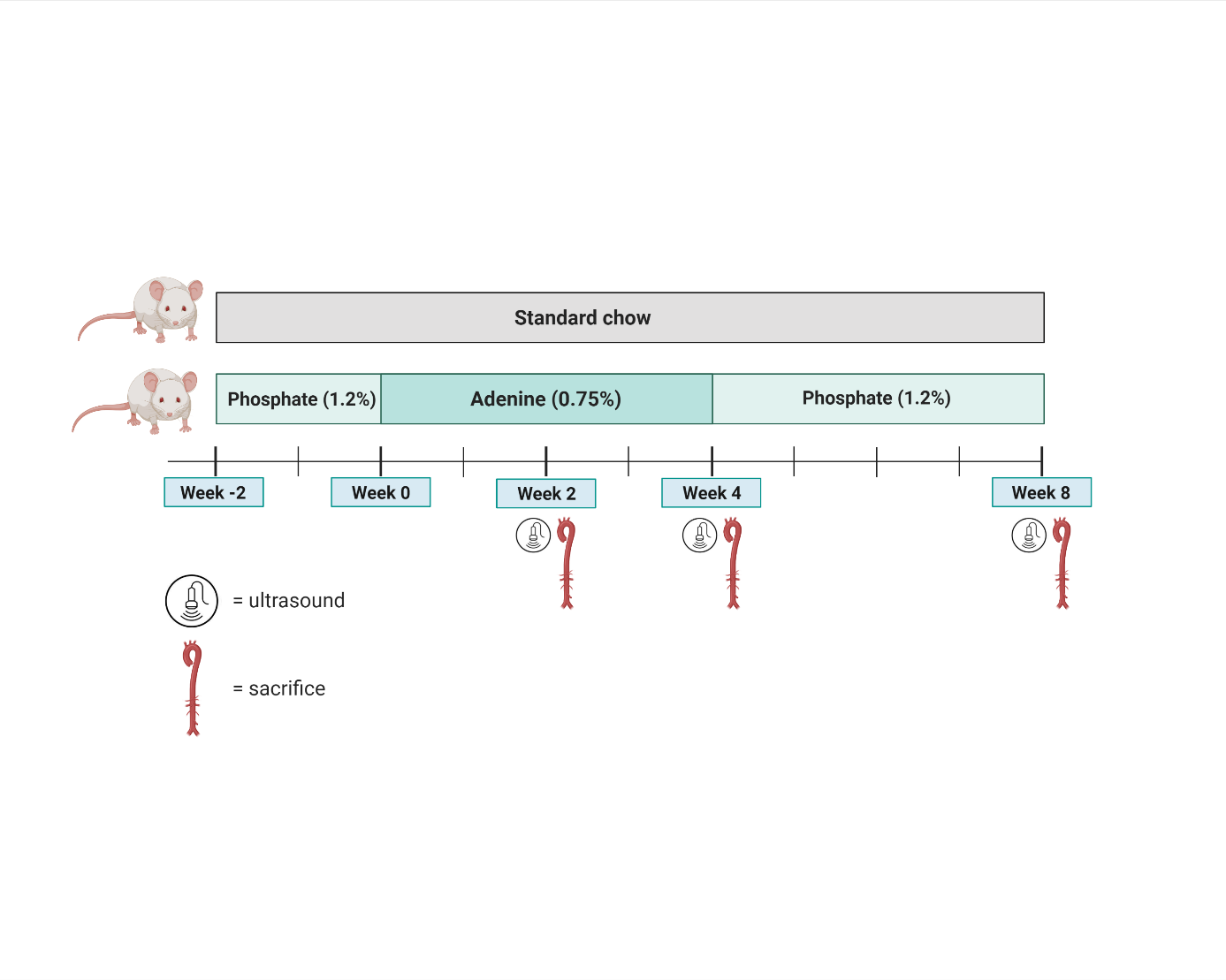


**Supplementary A.** Study overview: 44 male Wistar-Han rats (225–250g; Charles-River Laboratories, Belgium) were randomly assigned into different groups over different timepoints (2-, 4- and 8 weeks), consisting of (i) a control group with normal renal function and (ii) a CKD group which develops AMC. In the second group, CKD was induced by the administration of a high (1.2%) phosphate diet (Ssniff, Spezialdiäten, Soest, Germany) for 2 weeks followed by a 0,75% adenine-rich diet (Ssniff, Spezialdiäten, Soest, Germany) for 4 weeks. Animals of the 2- and 4 weeks group were respectively, sacrificed after 2- and 4 weeks of adenine treatment. Subsequently, animals of the 8 weeks group were fed by the high (1.2%) phosphate diet again for 4 weeks.


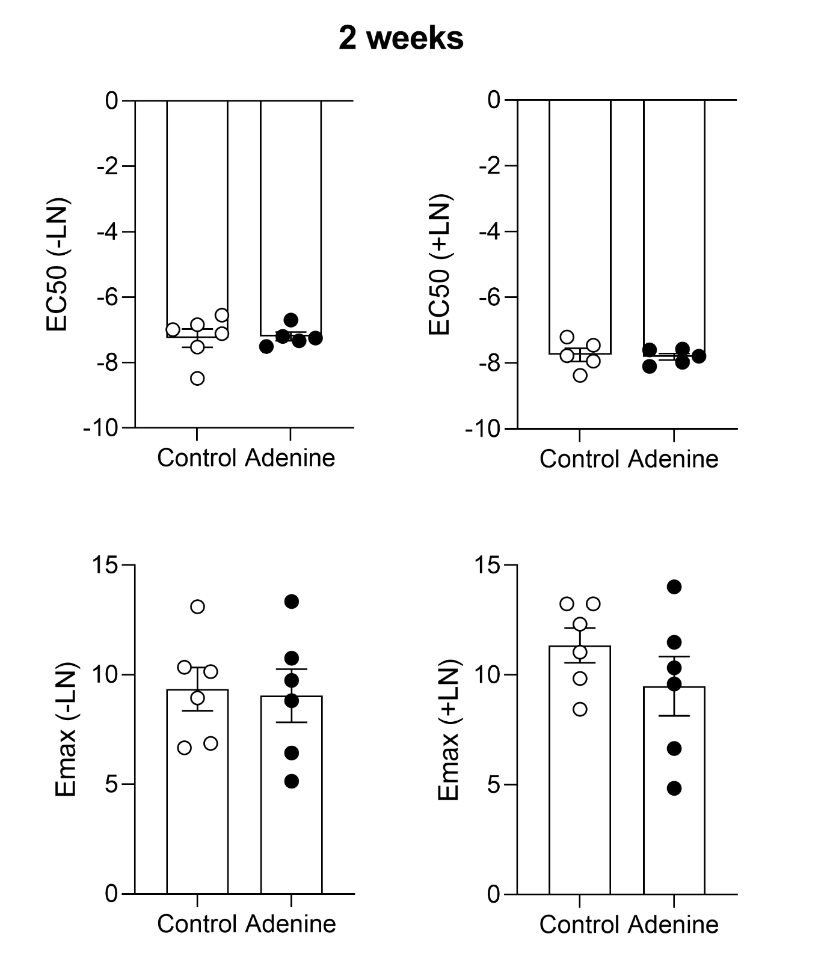


**Supplementary B.** Sensitivity (EC50) and maximal effect (Emax) upon phenylephrine(PE)-mediated VSMC contraction after 2 weeks of treatment. EC50 and Emax were calculated based on the concentration (3nM – 10μM) response curves in the presence and absence of N(ω)-nitro-L-arginine methyl ester (LN). Unpaired Mann-Whitney test (two-tailed): P > 0.05: not significant, not shown. Data represented as mean ± SEM.


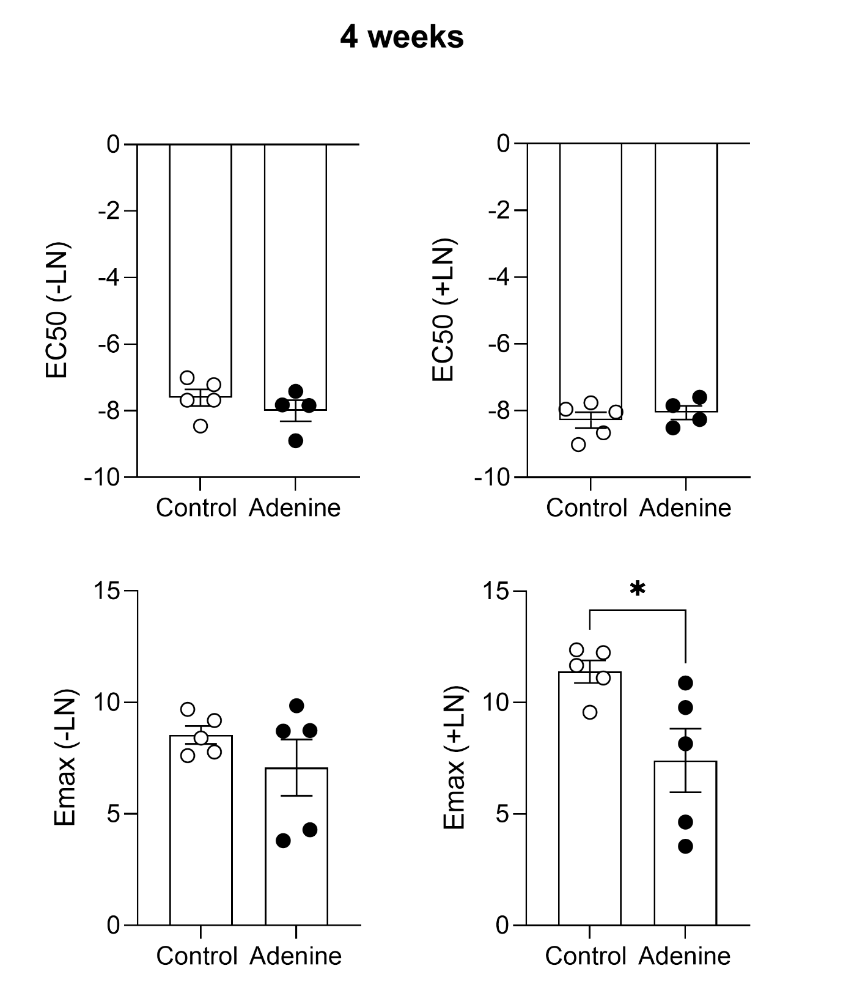


**Supplementary C.** Sensitivity (EC50) and maximal effect (Emax) upon phenylephrine(PE)-mediated VSMC contraction after 4 weeks of treatment. EC50 and Emax were calculated based on the concentration (3nM – 10μM) response curves in the presence and absence of N(ω)-nitro-L-arginine methyl ester (LN). Unpaired Mann-Whitney test (two-tailed): P > 0.05: not significant, not shown; P < 0.05: *. Data represented as mean ± SEM.
